# Dr. John Miller

**Published:** 1903-04

**Authors:** 


Dr. John Miller, of Buffalo, died at his residence, 48 St. John's Place, March 19, 1903, aged 79 years. He came to this country from Germany over fifty years ago and had practised medicine in western New York during that time, for the last twenty years in this city. He leaves a widow, three sons, and two daughters. His sons all are physicians,—Drs. John G. and Henry Miller residing at Lancaster and Dr. A. F. Miller, at Batavia.

